# Prevalence and predictors of viral load suppression in adults living with HIV in the western region of Ghana: A cross-sectional study

**DOI:** 10.3934/publichealth.2023033

**Published:** 2023-05-25

**Authors:** Philip Boakye, Adwoa Safowaa

**Affiliations:** 1 Department of Epidemiology and Biostatistics, Kwame Nkrumah University of Science and Technology, Kumasi, Ghana; 2 Faculty of Health and Medical Sciences, Presbyterian University, Agogo, Ghana; 3 Department of Research in Epidemiology, Biostatistics and Informatics, Koachie Health Systems, Accra, Ghana

**Keywords:** viral load, suppression, non-suppression, western region, adult HIV patient

## Abstract

**Background:**

Although antiretroviral therapy is beneficial and available free of cost to patients, several roadblocks still prevent patients from reaching viral suppression. This research aimed to determine the prevalence rate of viral suppression among people living with HIV in the western region of Ghana and identify the factors contributing to viral non-suppression.

**Methods:**

A cross-sectional study was conducted on 7199 HIV-positive adults. All data from the Sekondi Public Health Laboratory database was exported to Microsoft Excel and then verified and filtered before being exported to STATA 16.1. Viral non-suppression was modeled statistically using logistic regression.

**Results:**

Viral load suppression was achieved in 5465 (75.91%) study participants who received antiretroviral treatment. However, 1734 participants (24.0%) did not achieve viral suppression. Patients with poor adherence to ARV (AOR 0.30; 95% CI 0.16, 0.58) and fair adherence to ARV (AOR 0.23; 95% CI 0.12, 0.45) were associated with a lower odd of viral non-suppression. Patients with six (6) months to two (2) years of treatment before viral load testing (AOR 0.67; 95% CI 0.46, 0.98) were also associated with a lower likelihood of viral non-suppression.

**Conclusions:**

The rate of non-suppression was high, and the suppression rate fell short of the UNAIDS target. Poor ARV adherence, fair ARV adherence, and a treatment duration of six (6) months to two (2) years before viral load testing appear to be obstacles to viral load suppression. The research findings seem to suggest that viral load testing supports viral non-suppression. Therefore, using viral load tests to monitor medication's effects on health can motivate patients to adhere to their prescribed medication regimen. More research is needed to determine whether viral load testing can improve adherence. Given the high rate of virologic failure, the study highlights the importance of identifying antiretroviral resistance patterns.

## 1. Introduction

The HIV epidemic continues to pose a challenge to both public health and socioeconomic well-being [1]. Antiretroviral medication was available to 28.2 million patients in 2021. There were 37.7 million persons living with HIV in the world in 2020, of which 36 million were adults [2],[3]. AIDS has been identified as one of the leading causes of mortality among adults in sub-Saharan Africa [4]. According to a published report [3], the year 2020 witnessed 150,000 fatalities attributed to AIDS, while 3.5 million individuals sought medical intervention.

The World Health Organization defines viral suppression as viral load (VL) less than 1000 copies/ml and virological failure less than 1000 copies/ml [5],[6]. Viral failure or non-suppression is a consistently detectable viremia of more than 1000 copies/ml after a minimum of 6 months of antiretroviral therapy [6],[7]. To identify viral replication and confirm treatment failure, the World Health Organization recommends that people receiving antiretroviral therapy have their VL checked every six (6) months if possible [6],[8],[9].

Attaining a target of 90% of patients receiving antiretroviral therapy and achieving viral suppression was among the key goals of the United Nations Program on HIV/AIDS (UNAIDS) 90:90:90 initiative, which was slated to be accomplished by the year 2020 [10],[11]. However, this objective was not achieved due to the consequences of COVID-19, and the new global objectives are 95–95–95 by 2030 [12]. Globally, 47% of people living with HIV are virally suppressed, while the remaining 53% are not [10].

Ghana has an HIV population of 346,120 people as of 2020, with 19,267 new infections and 11,797 AIDS-related deaths [13]. HIV prevalence is 1.7% in the general population [13],[14]. Of the 346,120, 34% are male, 66% are female, and 245,223 were currently on treatment as of December 2021 [13]. To guarantee optimal viral load suppression, facilitate immunological reconstitution, limit HIV drug resistance development, avoid HIV-related mortality, and prevent treatment-resistant HIV variant transmission, patients with HIV need frequent HIV care and medication availability [15]–[17]. According to the 2020 estimate report, 25,620 people lived with HIV in the western region of Ghana. This represents 7.37% of the country's HIV population, and 18,930 people were on treatment in December 2021 [18].

Notwithstanding the increasing accessibility of free antiretroviral medication, several hindrances constrain the achievement of viral suppression. As per Ghana's 90–90–90 goal, the proportion of individuals who were aware of their HIV-positive status in 2020 was 64%, while 96% received antiretroviral therapy, and 73% achieved viral suppression [18]. Nonetheless, it is worth noting that there exists a dearth of reliable information pertaining to adults who are living with HIV and are either virally suppressed or not suppressed [19]. Furthermore, only a limited number of studies have been conducted to identify the predictors of non-viral suppression among adults residing in Ghana. The existence of research and data gap rendered it imperative to study and determine the prevalence rate of viral suppression and the factors associated with non-viral suppression.

## 2. Materials and methods

### 2.1. Study setting and data sources

A cross-sectional study was conducted at the Sekondi Public Health Laboratory (SPHL) from March 2020 to February 2021. This is the only testing laboratory in Ghana where viral load testing is done for the western and some parts of the western north regions. Each HIV-positive person who had been on antiretroviral therapy for at least six months and had sent blood samples to SPHL for VL testing between November 2019 and October 2020 was used as a data source. The study was done using records of 7199 patients.

Ethical clearance and approval for this study were obtained from Kwame Nkrumah University of Science and Technology, College of Health Sciences, School of Medicine and Dentistry, Human Research, Publication, and Ethics Committee (CHRPE). The reference number for the approval was CHRPE/AP/429/21. The entirety of the data provided by the study site was anonymous.

### 2.2. Eligibility criteria

#### 2.2.1. Inclusion

Patients who were on treatment for at least six months.

#### 2.2.2. Exclusion

Repeated test results were excluded, and only recent VL test results were included for each patient. Patients under 15 years of age were excluded from the research.

### 2.3. Sampling procedure

**Figure 1.**
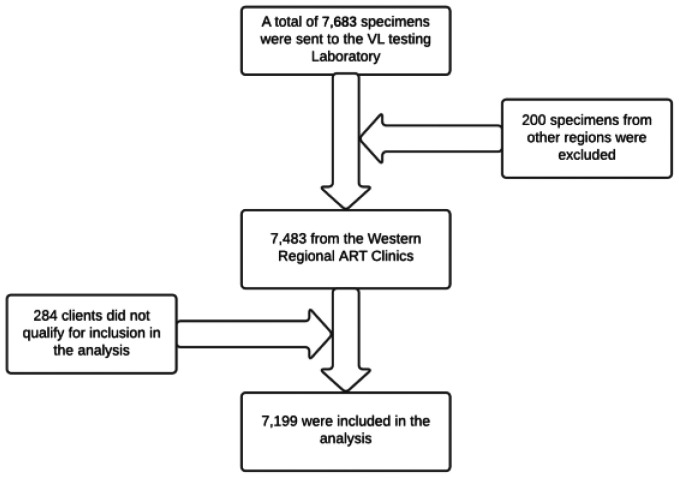
Schematic diagram of sampling procedures.

The data in the SPHL database were checked, and any samples collected from other regions were removed before analyzing the data. Finally, the study provided all the data that met the eligibility requirements (Figure 1).

### 2.4. Tools and methods for data collection

The HIV viral load data were retrieved from the SPHL database following a data extraction form. The following variables were included in the form: specimen identification (facility name, district), patient information (age, sex, pregnancy status, date of ART initiation, ARV adherence, and current regimen, and) indication for viral load testing (routine monitoring, viral load testing for repeat after detectable viremia and 3 to 6 months of enhanced adherence counseling and suspected treatment failure), and laboratory information (results). The viral load result was the dependent variable, part of the data extraction form. Before exporting to STATA 16.1, all data in the database were transferred to Microsoft Excel, and data verification and filtration were applied.

### 2.5. Assurance of data quality

In Microsoft Excel, the completeness and accuracy of the data were verified. The normality of the data set was confirmed using histogram plots and normal probability distributions. Controls were carried out before testing viral load samples at the SPHL.

### 2.6. Data analysis

The statistical analysis was conducted utilizing STATA 16.1. The classification of the result component of viral suppression status was based on the categorization of low- and middle-income countries by the World Health Organization. We measured the viral load (VL) test results in patients who had been on ART for at least six months. If the VL was less than or equal to 1000 copies/ml, it was considered viral non-suppression, while less than 1000 copies/ml was regarded as viral suppression. Adherence to medication was assessed through self-report, with good adherence defined as greater than or equal to 95%, fair adherence as between 85–94%, and poor adherence as less than 85% [5],[6],[20],[21]. The response variable was dichotomous, with a value of 1 indicating viral load not suppressed and a value of 0 indicating viral load suppressed. The study incorporated the following explanatory variables: age group, sex, facility type, facility ownership, district, pregnancy status, breastfeeding status, regimen, ARV adherence level, duration of treatment before viral load testing, and indication for viral load testing.

We used logistic regression to determine the relationship between the factors that affect viral load suppression. This statistical measure was expressed as an odds ratio (OR) with a 95% confidence interval. First, we conducted a bivariate analysis to assess the association between the independent and dependent variables. In the second step, we employed multivariable analysis, incorporating all variables into a single model. Finally, we included all significant variables at a P-value of ≤ 0.05 in the multivariable analysis into the final model. Our study utilized multivariable logistic regression to identify the factors independently associated with viral non-suppression.

## 3. Results

The research included a total of 7199 people. The median age of the research participants (IQR-interquartile range) was 43 (35–52) years. A suppression in viral load was observed in 5465 (75.91%) of the total research participants who received antiretroviral therapy. However, 1734 people (24.09%) did not achieve viral suppression.

**Table 1.** Characteristics of the Participants by Viral Load Suppression Status.

Variables	N (%)	Suppressed n (%)	Non-Suppressed n (%)
Age Category			
15–19	101 (1.40)	65 (1.19)	36 (2.08)
20–24	190 (2.64)	150 (2.74)	40 (2.31)
25–29	502 (6.97)	384 (7.03)	118 (6.81)
30–34	831 (11.54)	619 (11.33)	212 (12.23)
35–39	1159 (16.10)	873 (15.97)	286 (16.49)
40–44	1158 (16.09)	881 (16.12)	277 (15.97)
45–50	1205 (16.74)	928 (16.98)	277 (15.97)
>50	2053 (28.52)	1565 (28.64)	488 (28.14)
Gender			
Female	3690 (76.46)	2814 (76.97)	876 (74.87)
Male	1136 (23.54)	842 (23.03)	294 (25.13)
Facility Type			
Clinic	131 (1.82)	96 (1.76)	35 (2.02)
District Hospital	4282 (59.48)	3067 (56.12)	1215 (70.07)
Health Centre	656 (9.11)	458 (8.38)	198 (11.42)
Hospital	279 (3.88)	225 (4.12)	54 (3.11)
Regional Hospital	1851 (25.71)	1619 (29.62)	232 (13.38)
Facility Ownership			
CHAG	1202 (16.70)	829 (15.17)	373 (21.51)
Government	5773 (80.19)	4450 (81.43)	1323 (76.30)
Private	49 (0.68)	45 (0.82)	4 (0.23)
Quasi-Government	175 (2.43)	141 (2.58)	34 (1.96)
District			
Ahanta West	361 (5.01)	246 (4.50)	115 (6.63)
Ellembelle	885 (12.29)	651 (11.91)	234 (13.49)
Jomoro	460 (6.39)	359 (6.57)	101 (5.82)
Mpohor	5 (0.07)	5 (0.09)	0 (0.00)
Nzema East	263 (3.65)	167 (3.06)	96 (5.54)
Prestia Huni Valley	220 (3.06)	143 (2.62)	77 (4.44)
Sekondi-Takoradi	2954 (41.03)	2501 (45.76)	453 (26.12)
Shama	303 (4.21)	180 (3.29)	123 (7.09)
Tarkwa-Nsuaem	1052 (14.61)	716 (13.10)	336 (19.38)
Wassa Amenfi Central	6 (0.08)	3 (0.05)	3 (0.17)
Wassa Amenfi East	308 (4.28)	240 (4.39)	68 (3.92)
Wassa Amenfi West	331(4.60)	218 (3.99)	113 (6.52)
Wassa East	51(0.71)	36 (0.66)	15 (0.87)
Pregnancy Status			
No	1240(95.61)	879 (95.65)	361 (95.50)
Yes	57 (4.39)	40 (4.35)	17 (4.50)
Breastfeeding Status			
No	1327 (93.98)	944 (93.47)	383 (95.27)
Yes	85 (6.02)	66 (6.53)	19 (4.73)
Current Regimen			
DTG-based	2 (0.13)	2 (0.18)	0 (0.00)
D4T-based	6 (0.38)	4 (0.36)	2 (0.43
ABC-based	16 (1.01)	7 (0.63)	9 (1.92)
AZT-based	59 (3.72)	27 (2.42)	32 (6.84)
TDF-based	1503 (94.77)	1078 (96.42)	425 (90.81)
ARV Adherence Level			
Good	69 (4.18)	34 (2.97)	35 (6.93)
Poor	804 (47.09)	523 (45.68)	281 (55.64)
Fair	777 (48.73)	588 (51.35)	189 (37.43)
Duration on ART			
6–10 years	318 (18.49)	222 (18.32)	96 (18.90)
3–5 years	459 (26.69)	313 (25.83)	146 (28.74)
6 months–2 years	943 (54.83)	677 (55.86)	266 (52.36)
Indication for VL Testing			
Routine^a^	1972 (94.99)	1,422 (95.82)	550 (92.91)
Treatment failure^b^	10 (0.48)	5 (0.34)	5 (0.84)
Repeat^c^	94 (4.53)	57 (3.84)	37 (6.25)

*Note: ^a^Routine Monitoring; ^b^Suspected treatment failure; ^c^Repeat viral load test after detectable viremia and 3–6 months of enhanced adherence counseling. Abbreviations: CHAG = Christian Health Association of Ghana; ABC = Abacavir; AZT = Azidothymidine; TDF = Tenofovir Disoproxil Fumarate; DTG = Dolutegravir; D4T = Stavudine.

The bivariate analysis revealed a significant association between viral non-suppression and age category, regional hospital, facility ownership, Ellembelle, Jomoro, Sekondi-Takoradi, Shama and Wassa Amenfi East District, ABC, and AZT-based regimen, ARV adherence level and repeat viral load test.

However, in the final model, patients with poor adherence to ARV (AOR 0.30; 95% CI 0.16, 0.58), patients with fair adherence to ARV (AOR 0.23; 95% CI 0.12, 0.45) were associated with a lower chance of viral non-suppression. Patients with 6 months to 2 years of duration of treatment before viral load testing (AOR 0.67; 95% CI 0.46, 0.98) were also associated with a lower probability of non-suppression of the virus (Table 2).

**Table 2.** Predictors of Viral Non-Suppression among Adults Living with HIV.

Characteristic	OR (95% CI)	p-value	AOR (95% CI)	p-value
Facility Type				
Clinic	1		1	
District Hospital	1.08(0.73, 1.61)	0.678	1.46(0.60, 3.51)	0.402
Health Centre	1.18(0.78, 1.81)	0.428	1.67(0.67, 4.16)	0.274
Hospital	0.65(0.40, 1.07)	0.093	0.82(0.28, 2.44)	0.723
Regional Hospital	0.39(0.26, 0.59)	<0.001	0.44(0.17, 1.45)	0.093
ARV Adherence Level				
Good	1		1	
Fair	0.52(0.32, 0.86)	0.010	0.23(0.12, 0.45)	<0.001
Poor	0.31(0.19, 0.51)	<0.001	0.30(0.16, 0.58)	<0.001
Duration on ART				
6–10 years	1		1	
3–5 years	1.07(0.79, 1.47)	0.632	0.85(0.57, 1.27)	0.436
6 months–2 years	0.91(0.69, 1.20)	0.500	0.67(0.46, 0.98)	0.038
Indication for VL Testing				
Routine	1		1	
Treatment failure	2.59(0.75, 8.97)	0.134	2.11(0.13, 33.90)	0.599
Repeat	1.68(1.10, 2.57)	0.017	1.18(0.72, 1.96)	0.511

## 4. Discussion

The results reveal that 5465 individuals (75.91%) had viral suppression. Compared to the goal of the United Nations AIDS of 95% viral suppression, the proportion of viral suppression in this research is low [22]–[24]. It emerged from the analysis that viral non-suppression was 0.30 times more common in patients with poor adherence to antiretroviral treatment than in patients with good adherence (adjusted odds ratio [AOR] = 0.30, 95% confidence interval [CI]: 0.16, 0.58). Patients who had a fair adherence to their ARV treatment had a lower odd of viral non-suppression (adjusted odds ratio [AOR] of 0.23; 95 percent confidence interval [CI]: 0.12, 0.45). It was also observed that patients with six (6) months to two (2) years of duration on treatment before viral load testing were 0.67 times (odds ratio 0.67; 95% confidence interval (0.46, 0.98) likely to have viral non-suppression than those with six (6) to ten (10) years of duration on treatment. This study appears to be the first to find the factors associated with viral non-suppression among adult HIV-positive patients in the Western region.

These proportions of non-suppression among pregnant and breastfeeding women align with those obtained in a study in the Democratic Republic of the Congo [25]. The findings of this study suggest an even greater need to improve the quality of care for pregnant and breastfeeding women in Ghana. The male non-suppression rate of 25.13% is in line with those of [26]–[28], who found that males were more likely than females to be virally non-suppressed. Compared to research conducted in Ghana, the Ashanti region, in three ART clinics, the overall percentage of non-suppression was nearly the same as that found in this study [21]. Another study in Ethiopia discovered almost the same proportion of non-suppression [15]. Another research carried out in Ghana found that viral load non-suppression was very high [9]. The findings on the association between sex and viral load suppression status confirm those of earlier studies [21],[29]–[31] where there was no association between sex and viral load success or failure, just as this study did not find any association. Several other studies have also shown that low adherence to therapy is associated with long-term virological treatment failure [32]–[36]. This association is because when the concentration of medications in the blood decreases, HIV RNAs may not be suppressed, leading to an increase in the patient's viral load [15].

Additionally, it has been confirmed that people who remain on treatment and take their antiretroviral therapy have suppressed viral load [16]. However, a high VL indicates poor adherence to the medication [37],[38]. The results of the six (6) months to two (2) years duration of treatment before viral load testing are consistent with studies by [35],[39].

In contrast to the findings of [36],[40], this study did not find any association between age category and viral non-suppression. However, the age category was significant in the bivariate analysis. These results do not align with those of [36],[40], who found that the ART regimen was associated with viral non-suppression. Contrary to what has been reported by [21],[36], these results indicate no association between viral non-suppression and sex. Although [36] found that facility type has a significant association with viral non-suppression, this study's results suggest no association between facility type and viral non-suppression.

Although the study has its benefits, there are also some drawbacks to consider. The use of a single test result in the viral load study may not accurately show treatment failure. Additionally, the study did not take into account factors such as comorbidities, co-infections, educational level, treatment lines among HIV-positive individuals, and the potential for viral load testing to improve adherence. These limitations are important to remember when interpreting the study's results.

## 5. Conclusions

In summary, the research findings indicate a high rate of virological failure, with a suppression rate that falls below the 90% target set by UNAIDS. Possible obstacles to achieving viral load suppression were identified as inadequate adherence to antiretroviral (ARV) medication and a treatment duration ranging from 6 months to 2 years before viral load testing. The research findings appear to suggest that viral load testing supports viral non-suppression. Therefore, using viral load tests to monitor medication's effects on health can motivate people to adhere to their prescribed medication regimen. However, the research did not comprehensively clarify the factors that impact adherence. Additional research is required to investigate this topic more thoroughly and examine the potential of viral load testing to improve adherence. Furthermore, the study underscores the importance of determining the pattern of antiretroviral resistance in light of the substantial proportion of patients who did not achieve virologic suppression.
